# Reprohackathons: promoting reproducibility in bioinformatics through training

**DOI:** 10.1093/bioinformatics/btad227

**Published:** 2023-06-30

**Authors:** Thomas Cokelaer, Sarah Cohen-Boulakia, Frédéric Lemoine

**Affiliations:** Institut Pasteur, Université Paris Cité, Plate-Forme Technologique Biomics, 75015 Paris, France; Institut Pasteur, Université Paris Cité, Bioinformatics and Biostatistics Hub, 75015 Paris, France; Université Paris-Saclay, CNRS, Laboratoire Interdisciplinaire des Sciences du Numérique, 91405 Orsay, France; Institut Pasteur, Université Paris Cité, Bioinformatics and Biostatistics Hub, 75015 Paris, France; Institut Pasteur, Université Paris Cité, G5 Evolutionary Genomics of RNA Viruses, 75015 Paris, France

## Abstract

**Motivation:**

The reproducibility crisis has highlighted the importance of improving the way bioinformatics data analyses are implemented, executed, and shared. To address this, various tools such as content versioning systems, workflow management systems, and software environment management systems have been developed. While these tools are becoming more widely used, there is still much work to be done to increase their adoption. The most effective way to ensure reproducibility becomes a standard part of most bioinformatics data analysis projects is to integrate it into the curriculum of bioinformatics Master’s programs.

**Results:**

In this article, we present the *Reprohackathon*, a Master’s course that we have been running for the last 3 years at Université Paris-Saclay (France), and that has been attended by a total of 123 students. The course is divided into two parts. The first part includes lessons on the challenges related to reproducibility, content versioning systems, container management, and workflow systems. In the second part, students work on a data analysis project for 3–4 months, reanalyzing data from a previously published study. The *Reprohackaton* has taught us many valuable lessons, such as the fact that implementing reproducible analyses is a complex and challenging task that requires significant effort. However, providing in-depth teaching of the concepts and the tools during a Master’s degree program greatly improves students’ understanding and abilities in this area.

## 1 Introduction

In the past two decades, reproducibility has become a major concern in many disciplines, starting with social and psychological sciences (Open Science Collaboration 2015) and spreading to other domains such as pre-clinical research (Freedman et al. 2015), and computational and life sciences (Baker 2016; Cohen-Boulakia et al. 2017).

In wet biological experiments, there is generally an inherent variability due to the nature of the phenomena being examined, the methods of measurements, and the samples used. Conversely, it is common to assume that analyses in computational biology and bioinformatics are inherently reproducible due to the automated nature of their execution by machines. However, there are several factors that can hinder the reproducibility of analyses in computational biology and bioinformatics, including scientific obstacles like insufficient method documentation and data accessibility lacking the FAIR principles (Wilkinson et al. 2016) as well as technical challenges such as differences in operating systems and hardware (e.g. scheduler on HPC), disparate software environments (e.g. tools and libraries versions), and random algorithms.

To highlight the importance of reproducibility—and especially code sharing in this case—we can cite the discussions about BLOSUM matrices (Henikoff and Henikoff 1992) that were developed in 1992 to help for protein homology search and sequence alignments. Sixteen years later, Styczynski et al. (2008) corrected an issue in the BLOSUM source code, which performed worse than the original code. Eight years later, Hess et al. (2016) made new updates and showed that the new matrices performed better for homology search.

Over the last decade, several key scientific and technical advancements have been made. They have enabled computational biologists and bioinformaticians to design and implement highly complex data analyses with a higher degree of reproducibility [as defined in Cohen-Boulakia et al. (2017)] while also simplifying implementation and enabling easier maintenance and sharing of analysis code and results.

One notable example of these advancements is the development of workflow management systems such as Taverna (Oinn et al. 2004), the pioneer workflow system (which is no longer maintained) and more importantly Nextflow (Di Tommaso et al. 2017), Snakemake (Köster and Rahmann 2012), and Galaxy (Afgan et al. 2022) which are increasingly popular. These systems represent data analyses as “workflows,” in which the steps of the analysis are wrapped into processes that are connected to each other by data dependencies. Workflows have several benefits over traditional bash scripts. They allow analyses to be independent of the execution machine by removing the need for developers to implement code related to HPC schedulers. This makes the code more modular and easier to share and reuse. Additionally, workflow systems improve efficiency by implementing task parallelization. Overall, they facilitate the organization and coordination of the various components necessary for implementing and executing data analyses.

We can also emphasize the advancements made by virtualization technologies like Docker (Merkel et al. 2014) and Singularity (Kurtzer et al. 2017), which has recently been renamed Apptainer. For simplicity, we will continue to refer to it as Singularity hereafter. Docker and Singularity are software designed to make programs, daemons, servers, etc. executable on a large diversity of systems, and therefore to make the software environment more independent, shareable, easily executable, and maintainable. These two containerization engines thus made possible to encapsulate, share, install and execute any bioinformatics tools in an easy and lightweight way. When used in conjunction with workflow management systems, these container technologies facilitate the sharing and execution of complex data analyses.

Last, but not least, the use of code repositories such as GitHub and GitLab has greatly facilitated the collaborations and sharing of software and workflows. These platforms made possible the storage and sharing of large volumes of code, supporting the development and adoption of these technologies.

**Figure 1.**
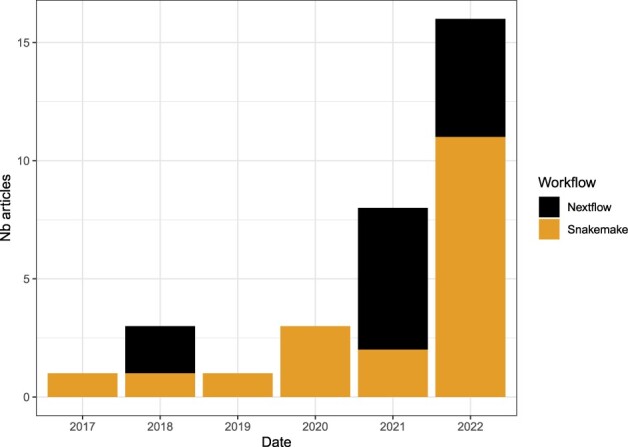
Number of articles in Nature or Science journals mentioning Nextflow and Snakemake, between 2017 and 2022, according to PubMedCentral search (10 January 2023).

As an illustration of the evolution of workflow usage in bioinformatic data analyses, an increasing number of publications are associated with newly implemented workflows (Haag et al. 2022), existing workflows (Grant et al. 2021), or at least public code (Tang et al. 2020) hence ensuring an easier way to reproduce the described results. For example, as shown in Fig. 1, searching for “Nextflow” or “Snakemake” in PubMed Central, in Science and Nature journals (therefore not specifically methodological journals) shows that: (i) workflows are more and more used (the number of papers is clearly increasing over the years), and (ii) there is still room for improvements (the maximum in 2022 is slightly over 15, which is still low). As more and more biological experiments are data intensive, we expect a continuous increase of data analyses in published papers, and hopefully an increase in usage of tools facilitating reproducibility.

However, despite their importance, these technologies are not always easy for researchers to learn and apply in their data analysis projects.

We strongly believe that bioinformaticians have to be systematically trained to reproducibility technologies and practices. In 2017, we started experimenting reproducibility hackathons in which groups of researchers (including PhD students, postdoctoral fellows, and more senior colleagues) were given an article published in a major place and had to try to understand how the main result of the article was obtained by reproducing it. A large variety of tools were used by participants including Python notebooks, workflow systems, or regular python scripts. These reproducibility hackathons took place from 2017 to 2019. They gathered between 10 and 15 participants each, around three different themes, namely next-generation sequencing, phylogenetics, and plant phenotyping. More information can be found at https://ifb-elixirfr.github.io/ReproHackathon/index-en.html.

This experience demonstrated how difficult it was to reproduce a published result, underlying how reproducibility is associated with various factors (choice of tools to implement a step of the analysis, choice of thresholds involved in statistical methods, environment of execution…), and how important it was to train a larger number of bioinformaticians.

We then leveraged this experience and created in 2020 the *Reprohackathon* class, in the Bioinformatics Master of sciences at Université Paris-Saclay (https://www.universite-paris-saclay.fr/en/education/master/bioinformatics/computational-biology/m2-biologie-computationnelle-analyse-modelisation-et-ingenierie-de-linformation-biologique-et-medicale) (France). The class has been running for 3 years now and has been attended by a total of 123 students (42 in 2020, 40 in 2021, and 41 in 2022). We designed it by including first lessons introducing the principles and tools necessary for applying the best practices in terms of reproducibility, followed by hands-on experience with these concepts and tools through a 3–4 months project.

Interestingly, in the meantime, several similar initiatives to promote reproducibility have been launched. Karathanasis et al. (2022) report feedback on a dedicated project involving seven students trained to reproduce a given scientific result (from a publication) using R/Python scripts. Millman et al. (2018) report one class of statistics where students were trained to reproduce Neuro-imaging results. Ostblom and Timbers (2022) describe how reproducibility has been included into a regular class to increase awareness on reproducibility. Last but not least, Ball et al. (2022) describe a Symposium of 10 lessons organized by the “UK reproducibility network” (https://www.ukrn.org/) on computational reproducibility with participants from various disciplines (linguists, political scientists, statisticians…).

Such initiatives are all complementary and globally conclude in the need to mix theoretical lessons and more hands-on projects. The challenge lies in coordinating the training effort and be able to systematically train the current and future generations of bioinformaticians to reproducibility. While coordination can be better achieved by the development of National networks on reproducibility [e.g. UK, Switzerland (https://www.swissrn.org/), Finland (https://www.finnish-rn.org/), Italy (https://www.itrn.org/)], we strongly believe that massively training students and colleagues means developing mandatory courses dedicated to reproducibility, involving full student classes, making explicit the reasons of lack of reproducibility, introducing major tools and technologies for computational reproducibility, mixing theoretical lessons, and large practical projects. The *Reprohackathon* course was designed to meet these needs and is the central focus of this publication.

This article is divided in three sections. The first section “Approach” describes in detail our *ReproHackathon* course from different perspectives: the profile of the students, the structure of the course, the expected difficulties, the content covered, the progress, and the evaluation. The second section, “Results”, describes students work and presents some student results in terms of reproducibility. The third section lists potential future directions in terms of scope, content, and audience and establishes guidelines for organizing *Reprohackathons*.

## 2 Approach

The objective of the *Reprohackathon* is 2-fold. First, it aims at introducing the concepts and tools the students need to create reproducible data analyses. The second goal is to give the students the opportunity to apply these skills through a hands-on project involving a RNA-seq data analysis pipeline.

### 2.1 Profile of the students

The *Reprohackaton* course was attended by 123 students enrolled in a Bioinformatics Master’s degree at Université Paris-Saclay. They graduated from various Bachelor degrees including computer science, statistics, and biology. They usually had no prior knowledge of high throughput technologies nor on reproducibility concepts and tools. They had varying levels of expertise, including students with no background in computer science and limited experience with Unix command line tools, to students with advanced knowledge of programming languages and command line tools. Similarly, their background in biology ranged from basic high school level understanding to current, advanced studies in the field. The diverse and interdisciplinary skills were valuable and exploited extensively during the class.

### 2.2 Lessons

The structure of the *Reprohackathon* was organized into two time periods. This subsection is focused on the first period which consists of lessons that provide a thorough introduction to the concepts, tools and technologies involved in reproducibility followed by 2 h slots of practical sessions where students were trained to use each of them in isolation.

**Figure 2.**
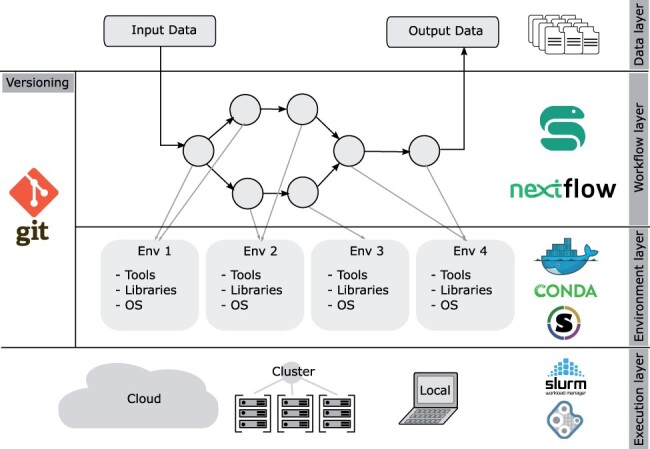
Outline of the analysis framework for achieving good reproducibility. From bottom to top, we define (i) the execution layer where the analysis steps will be executed, (ii) the environment layer, for managing software environment (i.e. bioinformatics tools, software, libraries, and operating systems), (iii) the workflow layer, for structuring the analysis as steps that call tools and consume and produce data, and (iv) the data layer, for handling the input and output data.

The initial lesson consists in a comprehensive overview of the principles of reproducibility, as well as an introduction to the issue of the reproducibility crisis and its growing significance in the field of bioinformatics. In addition, we provide an overview of best practices for implementing reproducible bioinformatics workflows within the framework we have defined (see Fig. 2).

The second lesson covers the importance of content versioning systems, with a focus on “Git”. The aim is to emphasize the importance of using “Git” or a similar system in any analysis, software, or script development, as it allows for more secure code development and the potential to share and collaborate with scientists worldwide. We then propose practical exercises using Git based on simple examples.

The third lesson focuses on the environment layer (see Fig. 2). It discusses the various existing systems for managing software environments, with a specific focus on Docker and Singularity [we also introduce other systems such as Conda with Bioconda (Grüning et al. 2018)]. Hands-on exercises are provided to help students build and run Docker and Singularity images for use with bioinformatic tools.

The fourth lesson is centered on the use of workflow management systems. We introduce them as orchestrators of the whole analysis, which make all layers work together, including execution, environment, workflow and data. In this lesson, we provide extensive instruction on Nextflow and Snakemake, as well as hands-on exercices for students to build basic workflows using these tools.

During the fifth and last lesson, we present the scientific project that will be the main focus of the second period (the *Reprohackathon per se*). The students form small groups of three or four individuals exploiting pluri-disciplinary skills and are given the choice of which workflow management system to use for the project, either Nextflow or Snakemake.

### 2.3 Subject of the project

During the second period, the *Reprohackathon* starts, during which the students apply the concepts and tools learned in the first period to a long-term project, which typically lasts for 3–4 months. During this project, the students work on a published dataset and attempt to analyze the data provided in the article. The goal is not to obtain exactly the same figures or results as the article, but rather to analyze the same dataset using similar, potentially more up to date methods, and determine the factors that are important for implementing a highly reproducible data analysis. In small groups, the students first have to read the papers and understand the methodology employed, the datasets, and the results. Then, they have to implement a reproducible workflow using either Nextflow or Snakemake for the workflow layer, Singularity or Docker for the environment layer, and Git for versioning and collaboration. Finally, they have to interpret the results obtained.

The datasets we proposed the students to work on were published in the two articles from Harbour et al. (2013) and Furney et al. (2013). In these papers, the authors are interested in the genetic determinants of uveal melanoma, a primary cancer of the eye that can result in fatal metastasis. We selected these two papers for three main reasons. First, publicly available datasets are provided in SRA (https://www.ncbi.nlm.nih.gov/sra) and ENA (https://www.ebi.ac.uk/ena/browser/), which allows easy reanalysis even almost 10 years after the publication. Second, these papers are adapted to our needs in terms of topic (RNA-Seq data analysis) and the experimental design is not too complex for the students (a simple comparison between two groups). Third, the data are large enough so that download and management is not straightforward and necessitate to think about implementation, but also not too large to be manipulated on the given machines (see Section 2.4) in terms of disk storage, memory and CPU usage, and computing time.

More precisely, Harbour et al. (2013) searched for specific mutations in uveal melanoma by exome sequencing of 18 primary tumors of class I (less invasive) and II (frequently metastasizes), and found deleterious mutations in two genes: *GNAQ* and *SF3B1* (Arginine to Cysteine in codon 626). The mutation in *SF3B1* was suggested to be associated with better prognosis. *SF3B1* encodes subunit 1 of the splicing factor 3b protein complex, which participates in splicing of pre-mRNAs. To analyze the effect of a mutation in this splicing involved protein, the authors analyzed samples in two ways: (i) they searched for differentially expressed transcripts between five mutants *SF3B1* and six wild-type *SF3B1* tumors using Illumina BeadArray platform and (ii) they analyzed 3 tumors with mutant *SF3B1* and 5 tumors with wild-type *SF3B1* using RNA sequencing. In (i) they found a few (10) differentially expressed genes, and in (ii) they found no signal for differential splicing. The second study, Furney et al. (2013) reanalyzed (among others) the RNA sequencing dataset of Harbour et al. (2013) and found that *SF3B1* mutations were associated with differential alternative splicing of protein coding genes such as *ABCC5* and *UQCC*, and of the long noncoding RNA *CRNDE*.

In our *Reprohackathon*, the students had to reanalyze this RNA sequencing dataset in order to look for (i) differentially expressed genes and (ii) differentially spliced genes (if they had time). The point (ii) was not a requirement, and was a bonus if they managed to get the point (i) done. To do so, they had to write a reproducible workflow using either Nextflow or Snakemake, with tools running either on Docker or Singularity containers. In the two first sessions (2020 and 2021), they did not have to build their own container images, and could use images already available on repositories such as DockerHub (https://hub.docker.com). In the last session (2022), they had to build their own container images.

**Figure 3.**
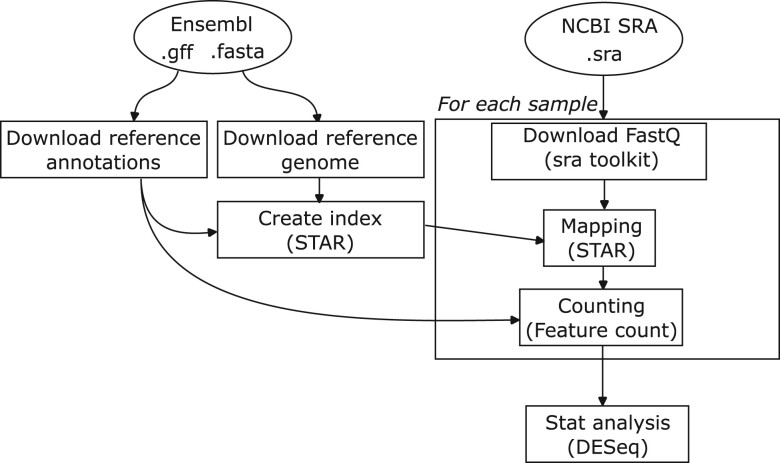
Workflow skeleton proposed to the students. It is made of about seven steps, from data download (reference + fastq) to read mapping and statistical analysis.

For differential gene expression, a skeleton workflow is provided to the students (shown in Fig. 3). It includes steps such as (i) downloads of the RNA sequencing data with sra-toolkit (https://github.com/ncbi/sra-tools) or equivalent software, (ii) download of reference genome sequence and annotations, (iii) creation of the genome index with STAR (Dobin et al. 2013), (iv) mapping reads onto the reference genome with STAR as well, (v) counting reads per gene using featureCounts (Liao et al. 2014), and (vi) analysis of differentially expressed genes with DESeq2 (Love et al. 2014). If time permits, students have also the possibility to analyze differentially spliced genes.

### 2.4 Computing resources

To develop and execute each workflow, the French Institute of Bioinformatics (*IFB* (https://www.france-bioinformatique.fr/)) provided us with a very large quota of CPU.hours on Biosphere (https://biosphere.france-bioinformatique.fr/), the federated cloud infrastructure maintained by *IFB*.

Biosphere proposes a large infrastructure (about 10 000 CPUs, 1.5Pb disk, and 40Tb cumulated RAM), distributed on many sites in France (Lyon, Clermont-Ferrand, Rennes, Nantes, Strasbourg and Lille), through a single interface. It comes with a large catalog of “Appliances” (already configured systems) that can be turned on with a chosen configuration ranging from small virtual machines (e.g. 1CPU, 8GB RAM, 20GB disk) to very large virtual machines (124CPUs, 3Tb RAM, 3Tb disk), the choice being dependent on the execution site.

For the *Reprohackathon*, students were given the possibility to turn on a virtual machine with an appliance already configured with the minimal requirements for running the project: conda (to install Singularity and Snakemake), Java (to install Nextflow), and Docker. From this minimal set of tools, everything can be executed.

### 2.5 Expected difficulties

We anticipated many technical and scientific challenges during the course, as the students have to get familiar with several concepts:

- Understanding the biology of the papers they were analyzing, which is more difficult for those with a computer science background.
- Rapidly becoming familiar with high throughput technologies, including the characteristics of RNA sequencing data and its biological meaning.
- Familiarizing themselves with common bioinformatic tools and concepts, such as public databases (SRA, Ensembl) and tools like STAR and DESeq2.
- Learning the basics of the Unix command line, including using SSH keys concepts to access remote computers and GitHub repositories.
- Managing the volume of sequencing data.
- Working with various programming languages and technologies, including Python, bash, R.

Also, even if the workflow is rather linear, there are several technical difficulties that are unavoidable in such workflows, such as the management of the paired-end FASTQ files together, or the meta data associated to each sample (mutated or wild-type) to be tracked throughout the workflow.

Last but not least, we expected technical difficulties inherent to any bioinformatics development, such as connection to distant linux machines, transfer of the files between local and distant machines (via Git or other means), installation of the required software, parameterizing the CPU, memory and disk quota requirements.

### 2.6 Progress

The *Reprohackathon* takes place over a period of three to four months. During this time, the students are expected to develop a high level of independence in their project management.

From the beginning of the *Reprohackathon* (in the second period, after the lessons), we meet with each group for 30 min on a weekly basis to offer guidance and support, similarly as if they were conducting a research project in a research unit. These meetings are initially focused on evaluating their understanding of the papers, troubleshooting technical issues, and helping the students to get started on their projects. As the students gain more autonomy, the meetings become less frequent. Overall, the goal is to provide the students with the opportunity to work independently, while still having support available when needed.

### 2.7 Evaluation

The expected learning outcomes are that students should be aware of the difficulty of building a reproducible and shareable data analysis, and they should know in theory and in practice the current concepts and tools they can apply to do so. Therefore, the students are evaluated on three aspects:

- The code. The code produced by a group must have been collaboratively developed using Git and a central repository (e.g. GitHub). It must be easily executable (by anyone), fully reproducible, and well documented. If they managed to get to the second part (alternative splicing), a bonus is granted.
- The report. It should describe the context of the project (biological: the published studies, and computational: the goal in terms of reproducibility), presenting the workflow they developed, providing the results they obtained by executing their workflow, and interpreting their results with regard to the given publications.
- An oral presentation during which they present the work of their group.

Since our goal is to put an emphasis on reproducibility, the code aspect had more weight in the final evaluation, as we estimated that these criteria were very important for the students to understand the benefits of writing code that is understandable and easily shareable.

## 3 Results and discussion

### 3.1 Workflow implementations

#### 3.1.1 Content versioning system

Workflows were implemented in a collaborative way, using Git and a central repository per group created by the students on GitHub.

**Figure 4.**
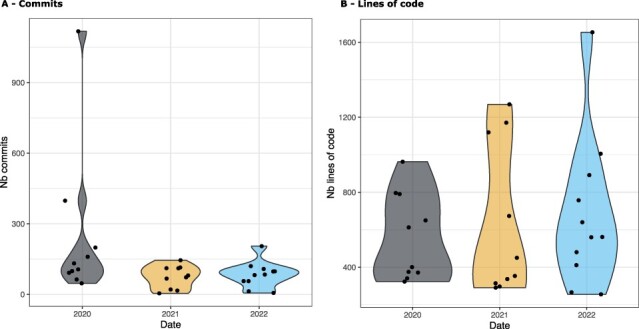
(A) Distribution of the number of commits per group and per year. The average number or commits is 241 for 2020, 74 for 2021, and 84 for 2022. There is 1 group in 2020 which is largely higher than the others, with more than 1000 commits. (B) Distribution of the number of lines of code by group. The average ranges from 562 to 680 between 2020 and 2022.

On average, students used Git effectively (see Fig. 4), with 241, 74, and 84 commits per group on average and 562, 627, and 680 lines of codes per group on average for the 2020, 2021, and 2022 sessions, respectively. The minimum and maximum number of commits were 4 and 1118, with 4 indicating an improper use of Git and 1118 indicating a potentially excessive use of commits and a too fine level of granularity.

Moreover, a few groups used the GitHub issues system in order to manage the progress of their projects, demonstrating an understanding of the usefulness of an online distributed content versioning system.

#### 3.1.2 Workflow languages

**Figure 5.**
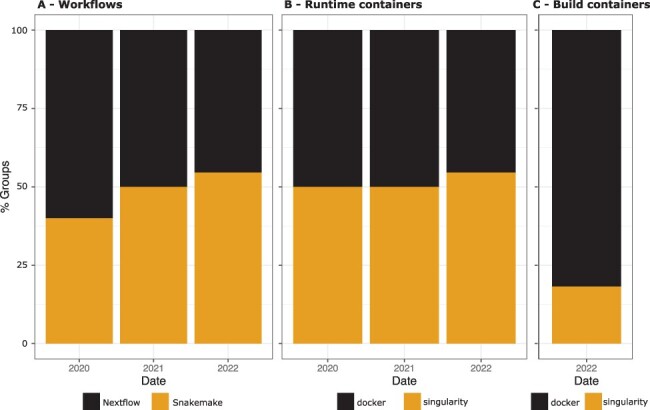
(A) Number of groups that chose Nextflow or Snakemake, per year. (B) Number of groups that chose Docker or Singularity for running the workflow, by year. (C) Number of groups that chose Docker or Singularity for building the containers (only asked in 2022).

In the *Reprohackathon* course, students were given the freedom to choose which workflow system they wanted to use for their project. The distribution of students choosing either Nextflow or Snakemake was generally well-balanced, with around 50% of students choosing each system (see Fig. 5A). Groups that chose Snakemake tended to do so because they were already comfortable with Python development, and thus thinking it would be advantageous. There was a slight increase in the percentage of groups choosing Snakemake over the years, which may be due to students having more training in Python during their university studies (web development, statistics, etc.).

Starting in 2022, we required to implement the workflows in DSL2 (https://www.nextflow.io/docs/latest/dsl2.html) (the latest Nextflow language) for Nextflow implementations. This led some groups to create highly modular Nextflow workflows, while Snakemake had already the mechanism implemented and was also used by some groups.

#### 3.1.3 Containers

The students had the choice of using either Singularity or Docker to build and execute container images to run their workflows.

Approximately half of the groups chose Docker over Singularity for running containers, as shown in Fig. 5B. This suggests that there is no clear advantage to using one technology over the other once the container is available (e.g. in DockerHub). It is important to note that the containers used in the first two sessions (2020 and 2021) were available on DockerHub, making it equally easy to use either Docker or Singularity. Both Snakemake and Nextflow support the ability to execute Singularity or Docker images from DockerHub through the use of Singularity command, which allows for a transparent conversion from DockerHub image to Singularity images.

Figure 5C shows that students preferred Docker for building containers in the 2022 session, during which students were asked to build their own images rather than use public ones. In a reproducibility perspective, it makes sense, since Docker images, once built, can be uploaded on DockerHub with almost no effort, and then be shared and executed by any instance of the developed workflow. This promotes reproducibility by ensuring that the workflow can be run consistently on any machine (provided that the DockerHub image retention policy allows for sufficient long-term storage).

In the 2022 session, students had to put a lot of effort into creating Docker and Singularity image recipes, which was a new challenge compared to previous sessions (2020 and 2021). This process involved learning the basics of installing Linux system packages, building packages from source, and understanding the concepts and syntax of image recipes. It took many attempts to get some of the images ready to run, especially for students with no computer science background. Overall, this was a valuable learning experience that gave students an insight into the work required to develop a project such as the *Reprohackathon*.

### 3.2 Workflow execution and re-execution

All the groups succeeded to execute their workflow on Biosphere (the *IFB* cloud), and when testing students implementations (re-execution), we tried to run their workflows on Institut Pasteur HPC cluster. This tested the ability of the developed workflow to run on a different environment (other hardware, operating system, file system, HPC task manager such as SLURM, etc.). After a few straightforward modifications of the workflow configurations (e.g. fix hard-coded filename, adapt to local SLURM executor), we managed to execute all the workflows. Re-execution times ranged from approximately 2–6 h, equivalent to approximately 25–98 CPU hours.

This large range in execution time may be due to a variety of factors, such as the differences in the configurations of the workflow in terms of CPU usage and memory (e.g. bad adequacy between CPU requirements and CPU allocated), more than the code itself. For example, some groups have used fasterqdump to download and convert FastQ data from SRA, while others used wget software to download the SRA files and fastqdump to convert them, which could be less efficient. Additionally, if the memory allocated for indexing the reference genome with STAR was insufficient, or if the reference genome was incomplete, the process could take longer to complete.

### 3.3 Workflow results

The execution of all student workflows was successful, and we were able to re-execute all of them. The results of these executions included lists of differentially expressed genes and various figures displaying the analysis of RNA-seq data (MA-Plots, PCAs, volcano plots). While we did not expect the results of the differential gene expression analysis to match those in the original papers due to variations in the analysis methods (e.g. software and annotations), we did expect the results across groups to be consistent. However, we were surprised to find that the results varied significantly.

**Figure 6.**
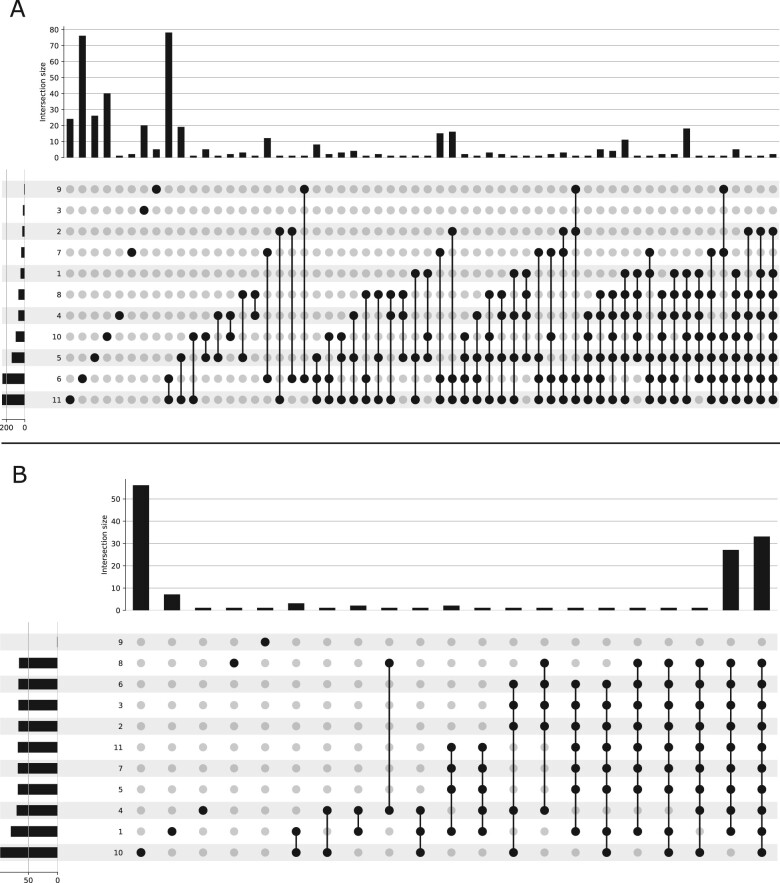
Comparison of differentially expressed genes among 2022 groups using upset plots. Histograms on the top of each upset plot represent the number of differentially expressed genes in common (and specific) to the groups indicated by circles below. Below the histograms, each row corresponds to a group. (A) Upset plot representing the number of differentially expressed genes in common between groups using students own statistical analysis. (B) Upset plot representing the number of differentially expressed genes in common between groups using homogeneous statistical analysis.

As a result, in the 2022 session, no differentially expressed genes were found in common between groups (see Fig. 6A). It is not surprising since at least one group took non-matching reference genome and annotations. Even though we exclude this group, the majority of the differentially expressed genes were common to only a few groups.

To understand the low reproducibility among groups, we divided the workflows into two parts: (i) The part from data download to read counting, and (ii) the part using read counts to perform statistical analysis with DESeq2. This allowed us to identify several factors contributing to this variability. Firstly, the factors related to the first part of the workflow, such as workflow code errors, were found to be a significant source of variability. For example, we observed groups with consistent read counts, but associated with the incorrect samples (mislabeled columns). Secondly, factors linked to tools and data usage like not using the same version, not having the same tool parameters, not using the same reference genome, or using the wrong data, can also contribute to variability despite using container technologies. An example of this is a group that downloaded a version of the human genome annotations that was not compatible with the version of the human genome they were using. Also, several groups did not download all the chromosomes (in particular, mitochondrial genome was sometimes missing). Third, other factors linked to the statistical analysis (second part of the workflow consisting of a R script) were more important: (i) The choice of thresholds such as p.value, fold change, (ii) filtering of lowly expressed genes, (iii) the implementation of the experimental design (which sample to assign to which condition, etc.), and (iv) the choice to exclude a sample based on quality control. Default parameters of the statistical analysis were also tuned differently in some groups as summarized in Table 1.

**Table 1.** Main differences between groups.

Group	Ref	DeSeq2 filters and options	padj	l2FC	Paired (fc)
1	hg38	Gene-count based	0.05	0	Yes
2	hg38	LRT	0.01	0	Yes
3	hg38	Default	0.05	0	No
4	hg38	Default	0.05	1.5	Yes
5	hg38	Gene-count based, shrinkage	0.1	0	No
6	hg38	Gene-count based	0.05	0	Yes
7	hg38	Gene-count based	0.05	1	No
8	hg38	Gene-count based	0.05	0	No
9	hg38	Gene-count based, LRT	0.05	1.5	Yes
10	hg37	Default	0.05	0	No
11	hg38	betaPrior, outlier removed	0.1	0	Yes

The column *ref* indicates whether hg37 or hg38 was used as reference. The column *DeSeq2* indicates specific options or filters applied before statistical analysis; *gene-count based* means that a filter was applied to exclude genes (e.g. minimal number of reads across all conditions was required) *LRT* (log likelihood test) and *shrinkage* are options related to DeSeq2 analysis. The *padj* and *l2FC* columns gives the adjusted *P*-value and log2 fold change thresholds used to select the final list of differentially expressed genes. The last columns indicates whether the paired option was added in the option at the feature count level.

We then looked at the reproducibility of the first part of the workflow by taking read count matrices from all groups results, and apply the same analysis (our own). In that case, we managed to obtain a very high number of differentially expressed genes in common across the groups. On Figure 6B, we can see that two groups stand out from the others. The first one (10) outputs a huge number of differentially expressed genes compared to the others, and hence a lot of them are found only by this group. However, group 10 finds many genes in common with the other groups. The second group (9) is also very different from the others, because they found very few differentially expressed genes, all being specific to that group. If we take these two groups apart, (9 and 10), most of the genes are found in common, which shows that most of the variability comes from the second part of the workflow.

After the differential gene expression, over the past 3 years, two groups (in 2020) managed to get to the alternative splicing analysis (unintuitively, lockdowns may have been beneficial for projects in autonomy). One group used DEXSeq (Anders et al. 2012) to count reads on exons and to extract genes that have a differential exon usage between both conditions, and the other group simply counted reads on all exons and then applied a classical differential gene expression analysis. As a result, they did not find genes in common. This case shows again that studies are always hard to reproduce, and that many factors may make the results variable.

### 3.4 Student feedbacks

We sent the students a survey to collect their feedbacks from all three sessions of this program. We obtained a total of 47 respondents (39%). About 80% of the respondents followed the class in the last 2 years. Students registered in 2020–2021 constituted only 20% of the answers. This survey shows that they generally enjoyed the course: 77% enjoyed it or enjoyed it a lot, 12% of the students enjoyed it only a little and 8% not at all, 4% have no opinion. Students expressed positive feedback about the reproducibility awareness, 85% of respondents estimate that this program made them aware of reproducibility issues, 89% reported that they may apply the concepts they have learned during the program in their future activity. Regarding the difficulty, 47% estimate that the program is difficult and another 47% estimate it has an expected difficulty. The most difficult part seems to be the usage of workflows (49%), followed by containers (25%). Regarding the skills, 71% (resp. 47%) declared that they acquired (very) strong knowledge in workflow systems (resp. Git).

Some points were raised in a free text feedback form: (i) Git and Linux commands could be taught and be exploited more deeply, (ii) too many technologies were introduced and the practical sessions before the project could be longer, and (iii) the project was a little too ambitious. Some of these points were already raised before the survey and have been taken into account to adapt the course over the years.

### 3.5 Faced challenges and main messages

To summarize students feedback and our own, students essentially faced three different types of challenges during the *Reprohackathons* (see Section 2.5 for details): technical, scientific, and project management.

For the technical challenges, students had to learn various technological stacks, such as Unix Shell and workflow management, which sometimes led to unexpected confusion or misunderstandings. For example, errors in CPU or memory allocation occurred when students tried to determine the number of CPUs on the execution machine by reading system files (/proc/cpuinfo).

For the scientific challenges, for some students it was the first time that they had to work on such a multi-disciplinary project, in which they had to be proficient in biology, bioinformatics, and statistics. Moreover, from a reproducibility perspective, students encountered a real challenge, and realized the importance of containerization but also choices to be made on filtering the data, parameters, software versions, etc.

For the project management challenges, students had to organize their time during a long period (∼3 months), without necessarily initially realizing the amount of work the project would necessitate.

The main messages we tried to convey during these *Reprohackathons* are the following:

1. Developing a reproducible data analysis workflow is hard. First, many choices are (often not explicitly) made during the course of a project. Then, it is difficult to implement the reproducible analysis using available tools.
2. Documentation is key: with the same initial information, different interpretations were made, leading to different results. For example: (i) one sample was ambiguous in its assignment as a mutated or wild-type. (ii) some groups filtered low counts differently. (iii) groups did not take the same (adjusted) *P*-value threshold. Put together, PCA plots, and gene lists varied significantly.

## 4 Future of the *Reprohackathons* and guidelines

In this section, we describe the next improvements planned for the *Reprohackathon* course in the next years and we then list a set of lessons learned from this project, hoping to provide useful guidelines to anyone aiming to run a similar course.

### 4.1 Future directions

We have already run *Reprohackathons* for 3 years and we will continue to do so. On a pure organization point of view, our setting appears to be really satisfying in particular (i) the time span (3–4 months at a rate of once a week) which is well adapted to the mix of lessons and project, and gives time for student to practice a lot; (ii) the format with several hours of work for students outside the course sessions and regular meetings providing autonomy to the students and favorable to learning by practice.

We plan to make four changes in the next year. First, on an organizational point of view, we will have two periods in the project: a competitive phase (between groups) as usual, followed by a collaborative phase, allowing students to compare their results and better understand the factors of variability of the study. Second, on a technical point of view, we will explore other ways of using virtual machines (based on remote desktops for example). This will circumvent classical issues for students (e.g. connect to the virtual machines, make file transfer for execution, configuration of SSH and GitHub keys in the integrated development environments, etc.). Third, on an evaluation point of view, we will add more stringent criteria to compare the results obtained by students with the results of the papers and to compare results of students. Fourth, on a content point of view, we will consider new studies both because results of the previous *Reprohachathons* are available on GitHub (and we want to avoid cheating) and because we aim to explore other domains and other data types to analyze. We are investigating a study of viral sequencing data (e.g. SARS-CoV-2), from sequence data analysis to phylogenetics, as another nice example of the importance of reproducibility.

Looking further ahead, we plan to help in setting up the *Reprohackathons* in other Master’s programs. In this regard, we already discussed with colleagues from Université Paris Cité who participated to the defenses of the 2022 session. We are convinced that a course such as our *Reprohackathon* should be mandatory in any Master of science curriculum. More generally, we are working on setting-up a website to collect and share training material on reproducibility. We are currently also working on providing a set of videos on the key tools and technologies identified and addressed during the lessons part of the *Reprohackathon*.

### 4.2 Guidelines

We identified several points that form the basics of the organization of *Reprohackathons*, important for a successful implementation.

*Take enough time for lessons*. Initial lessons and practices about the importance of reproducibility and the tools and technologies that will be used in the project (git, Conda, Docker, Snakemake, Nextflow, etc.) are fundamental. It gives the students a basic knowledge that will allow them to dig further autonomously when needed.

*Choose appropriate papers*. The analysis we ask the students to work on is a major part of the project. First of all, the paper should focus on a scientific problem involving bioinformatics data processing. It may be sequencing data analysis, methodological developments, tool benchmarks, etc. It should present a structured bioinformatics analysis with clear results, and feasible according to the available computational resources. Last but not least, the analyzed data must be publicly and easily accessible, with unique identifiers (close to the FAIR principles). Contacting authors is also an important point of the process: the aim of a *Reprohackathon* is not to blame and shame studies’ authors but rather to promote the need for more reproducibility by showing how easy it is to have variability in the results of a study.

*Define the goals*. As it may be their first long term project requiring such an autonomy, the goals of the *Reprohackathon* are not always clear for the students in the beginning. It is important to clarify quickly what is expected from the students in terms of reproducibility and code. To do so, it is important to delineate the subject in terms of workflow (what is the expected kind of workflow output), but also in terms of tools (what kind of tools should be integrated in the workflow). In the three last sessions, we decided to propose two milestones: (i) differential gene expression, and (ii) differential splicing, for which we defined the tools students may use. The second milestone was optional, and considered as a bonus.

*Test the workflow in advance*. It is important to implement and test the workflow before the session starts, to be sure that (i) datasets are effectively available, (ii) the workflow is realizable, and (iii) results are interpretable and reproducible. It will limit the number of surprises during the project.

*Define the evaluation criteria*. The evaluation criteria, linked to the previous point, are very important to clearly define. For the three last sessions, we focused on the code, the documentation, the repeatability of the produced workflows on a different environment, and the results. For the next sessions, we may add more stringent criteria on the proximity of the results with the results of the papers.

*Form diverse multidisciplinary working groups*. Groups of students should be as diverse as possible in terms of skills. Groups mixing students with biology background with computer science and/or statistical background have proved to be very efficient.

*Be aware of simultaneous running of complementary courses*. The 2022 session of the *Reprohackahton* took place in the same period than other complementary courses, such as “Next Generation Sequencing data analysis”. This helped the students a lot to integrate the many concepts they needed to advance the project. In addition, students took advantage of the situation by asking many questions to other course professors (statistical questions or RNA-Seq related questions, etc.), thus serving as mentors for these parts.

*Access to computational resources*. Computational/IT resources constitute another critical aspect. First of all, their sizing must correspond to the needs of the analysis to reproduce. In addition, they must be flexible enough to allow to start and stop the machines as necessary based on need. In the last three sessions, we chose not to use a proper cluster (with a scheduler such as SLURM for example), as it would have added an additional technological stack to introduce. However, many possibilities are offered nowadays: public clouds (e.g. Biosphere), private clouds, clusters, or even local machines if the study to reproduce in not too memory or CPU intensive. In the case of institutional clouds or clusters, it may be subjected to a request for support, which may take some time and possibly dedicated funding.

*Encourage collaborative work between groups*. We noticed that many groups collaborated with each others during the project. It was generally about sharing their difficulties and their solutions, but rarely to share large part of their code. We realized these collaborations were highly fruitful, since it helped them avoid a few traps. On this matter, next sessions of *Reprohackathon* will have two periods in course of the project: competitive as usual and collaborative, allowing students to compare their results and better understand the factors of variability.

*Regular meetings*. We observed that organizing small individual meetings with each group individually on a weekly basis was very efficient and appreciated by the students. On the one hand, it gave the students the autonomy to organize their work as they wanted, and in the other hand, the meetings were the occasion to be focused on groups individually, and to discuss clear progress reports.

*Clear deliverables*. We think that, as usual with university projects, the main deliverables should be: the code, a report manuscript, and a final presentation. A noteworthy outcome that we reported in the preceding sections was the disparities in results observed across the groups. We think that it would be valuable to organize a last feedback session after the final presentation, where students can share their results and try to understand the origin of the disparities.

*Evaluation committee*. Inviting external members to the defense adds a lot to the final oral presentations. They usually have complementary questions that give good ideas for future sessions.

## 5 Conclusion

In this article, we presented the *Reprohackathon*, a Master’s course we have been running at Université Paris-Saclay since 2020. The goal of the *Reprohackathon* is to make students aware of major reproducibility difficulties, and to promote good practices for designing and executing bioinformatics data analyses.

The originality of our approach lies in developing a mandatory course dedicated to reproducibility, involving a full class of students, making explicit the reasons of lack of reproducibility, introducing major tools and technologies for computational reproducibility defined through different layers, and mixing theoretical lessons and a large practical project. The *Reprohackathon* takes the form of a 3- to 4-month project during which students (in groups) work on a scientific article involving a bioinformatic data analysis. They have to (i) understand the paper, (ii) understand and implement the data analysis workflow, and (iii) analyze the data provided in the paper. The implemented workflow must be reproducible and shareable, such that we can easily reproduce the results on a different environment.

As a result, the comparison of project outputs across groups showed a high variability caused by several factors: alternative version of the reference dataset, alternative tools used to implement a given step of the analysis, various choices of cutoffs, impact of the execution environment to make the various tools orchestrate correctly. This allowed us to make explicit for students all the factors playing a role in reproducibility.

More generally speaking, our aim is to promote reproducibility and provide assistance to colleagues who would be interested in running the same kind of course. Based on our experience, we established a set of guidelines to help organize *Reprohackathons* in the best conditions. As future work, we plan to set-up a website to collect and share training material on reproducibility. We are currently working on a set of videos to make available for the bioinformatics community to support training on the key tools and technologies identified and addressed during our lessons.

## Data Availability

The data and scripts used to produce the figures are available on GitHub at https://github.com/fredericlemoine/reprohackathon_ismb2023.

## References

[btad227-B1] Afgan E , Nekrutenko A, Grüning BA et al The galaxy platform for accessible, reproducible and collaborative biomedical analyses: 2022 update. Nucleic Acids Res 2022;50:W345–51. 35446428 10.1093/nar/gkac247 PMC9252830

[btad227-B2] Anders S , Reyes A, Huber W et al Detecting differential usage of exons from RNA-seq data. Nat Prec 2012;22:2008–17. 10.1101/gr.133744.111 PMC3460195 22722343

[btad227-B3] Baker M. 1,500 scientists lift the lid on reproducibility. Nature 2016;533:452–4. 27225100 10.1038/533452a

[btad227-B4] Ball R , Medeiros N, Bussberg NW et al An invitation to teaching reproducible research: lessons from a symposium. J Stat Data Sci Educ 2022;30:209–18.

[btad227-B5] Cohen-Boulakia S , Belhajjame K, Collin O et al Scientific workflows for computational reproducibility in the life sciences: status, challenges and opportunities. Future Gener Comput Syst 2017;75:284–98.

[btad227-B6] Di Tommaso P , Chatzou M, Floden EW et al Nextflow enables reproducible computational workflows. Nat Biotechnol 2017;35:316–9. 28398311 10.1038/nbt.3820

[btad227-B7] Dobin A , Davis CA, Schlesinger F et al Star: ultrafast universal RNA-seq aligner. Bioinformatics 2013;29:15–21. 23104886 10.1093/bioinformatics/bts635 PMC3530905

[btad227-B8] Freedman LP , Cockburn IM, Simcoe TS et al The economics of reproducibility in preclinical research. PLoS Biol 2015;13:e1002165. 26057340 10.1371/journal.pbio.1002165 PMC4461318

[btad227-B9] Furney SJ , Pedersen M, Gentien D et al Sf3b1 mutations are associated with alternative splicing in uveal melanoma. Cancer Discov 2013;3:1122–9. 23861464 10.1158/2159-8290.CD-13-0330 PMC5321577

[btad227-B10] Grant RA , Morales-Nebreda L, Markov NS et al; NU SCRIPT Study Investigators. Circuits between infected macrophages and T cells in SARS-CoV-2 pneumonia. Nature 2021;590:635–41. 33429418 10.1038/s41586-020-03148-w PMC7987233

[btad227-B11] Grüning B , Dale R, Sjödin A et al; Bioconda Team. Bioconda: sustainable and comprehensive software distribution for the life sciences. Nat Methods 2018;15:475–6. 29967506 10.1038/s41592-018-0046-7 PMC11070151

[btad227-B12] Haag J , Höhler D, Bettisworth B et al From easy to hopeless—predicting the difficulty of phylogenetic analyses. Mol Biol Evol 2022;39:msac254. 36395091 10.1093/molbev/msac254 PMC9728795

[btad227-B13] Harbour JW , Roberson EDO, Anbunathan H et al Recurrent mutations at codon 625 of the splicing factor SF3B1 in uveal melanoma. Nat Genet 2013;45:133–5. 23313955 10.1038/ng.2523 PMC3789378

[btad227-B14] Henikoff S , Henikoff JG. Amino acid substitution matrices from protein blocks. Proc Natl Acad Sci USA 1992;89:10915–9. 1438297 10.1073/pnas.89.22.10915 PMC50453

[btad227-B15] Hess M , Keul F, Goesele M et al Addressing inaccuracies in blosum computation improves homology search performance. BMC Bioinformatics 2016;17:1–10. 27122148 10.1186/s12859-016-1060-3 PMC4849092

[btad227-B16] Karathanasis N , Hwang D, Heng V et al Reproducibility efforts as a teaching tool: a pilot study. PLoS Comput Biol 2022;18:e1010615. 36355750 10.1371/journal.pcbi.1010615 PMC9648701

[btad227-B17] Köster J , Rahmann S. Snakemake—a scalable bioinformatics workflow engine. Bioinformatics 2012;28:2520–2. 22908215 10.1093/bioinformatics/bts480

[btad227-B18] Kurtzer GM , Sochat V, Bauer MW et al Singularity: scientific containers for mobility of compute. PLoS One 2017;12:e0177459. 28494014 10.1371/journal.pone.0177459 PMC5426675

[btad227-B19] Liao Y , Smyth GK, Shi W et al Featurecounts: an efficient general purpose program for assigning sequence reads to genomic features. Bioinformatics 2014;30:923–30. 24227677 10.1093/bioinformatics/btt656

[btad227-B20] Love MI , Huber W, Anders S et al Moderated estimation of fold change and dispersion for RNA-seq data with DESeq2. Genome Biol 2014;15:1–21. 10.1186/s13059-014-0550-8 PMC4302049 25516281

[btad227-B21] Merkel D et al Docker: lightweight linux containers for consistent development and deployment. Linux J 2014;239:2.

[btad227-B22] Millman KJ , Brett M, Barnowski R et al Teaching computational reproducibility for neuroimaging. Front Neurosci 2018;12:727. 30405329 10.3389/fnins.2018.00727 PMC6204391

[btad227-B23] Oinn T , Addis M, Ferris J et al Taverna: a tool for the composition and enactment of bioinformatics workflows. Bioinformatics 2004;20:3045–54. 15201187 10.1093/bioinformatics/bth361

[btad227-B24] Open Science Collaboration. Estimating the reproducibility of psychological science. Science 2015;349:aac4716. 26315443 10.1126/science.aac4716

[btad227-B25] Ostblom J , Timbers T. Opinionated practices for teaching reproducibility: motivation, guided instruction and practice. J Stat Data Sci Educ 2022;30:241–50.

[btad227-B26] Styczynski MP , Jensen KL, Rigoutsos I et al Blosum62 miscalculations improve search performance. Nat Biotechnol 2008;26:274–5. 18327232 10.1038/nbt0308-274

[btad227-B27] Tang J , Fewings E, Chang D et al The genomic landscapes of individual melanocytes from human skin. Nature 2020;586:600–5. 33029006 10.1038/s41586-020-2785-8 PMC7581540

[btad227-B28] Wilkinson MD , Dumontier M, Aalbersberg IJ et al The fair guiding principles for scientific data management and stewardship. Sci Data 2016;3:1–9. 10.1038/sdata.2016.18 PMC4792175 26978244

